# The Preliminary Study of Water-Retention Related Properties of Biochar Produced from Various Feedstock at Different Pyrolysis Temperatures

**DOI:** 10.3390/ma12111732

**Published:** 2019-05-28

**Authors:** Koji Kameyama, Teruhito Miyamoto, Yukiyoshi Iwata

**Affiliations:** Institute for Rural Engineering, National Agricultural and Food Research Organization, 2-1-6 Kannondai, Tsukuba, Ibaraki 305-8609, Japan; teruhito@affrc.go.jp (T.M.); iwatayuk@affrc.go.jp (Y.I.)

**Keywords:** biochar, slow pyrolysis, waste biomass, soil amendment, water-retention, pore-size distribution

## Abstract

Physicochemical properties of biochar, which are used as a soil amendment material in agricultural fields, are different depending on biomass feedstock and pyrolysis processes. In this study, we evaluated the influence of feedstock type and pyrolysis temperature on the water-retention related properties of biochar. Wood-chips [cedar (CE) and cypress (CY)]; moso bamboo (MB); rice husk (RH); sugarcane bagasse (SB); poultry manure (PM) and agricultural wastewater sludge (WS) were each pyrolysed at 400, 600 and 800 °C with a retention time of two hours. Scanning electron microscopy micrographs (SEM), hydrophobicity indices, pore-size distribution measured by mercury-intrusion porosimetry, water-retention curves (WRCs) and plant-available water capacities (AWCs) of the biochars were measured to evaluate their potentials as soil-amendment materials for improving soils’ water-retention. As the pyrolysis temperature was increased, the hydrophobicity index decreased. On the other hand, pyrolysis temperature did not affect the distribution of micrometre-range pores, which are useful for plant-available water, of biochars. The AWCs of the biochars formed from CE, CY and SB were greater than those produced from other feedstocks, at 600 and 800 °C. Therefore, we can suggest that the biochars derived from wood-chips (CE and CY) and SB have greater potential for enhancing soils’ water-retention.

## 1. Introduction

Biochar is the solid product obtained from thermochemical conversion of biomass in an oxygen-limited environment [1]. Biochar can be used as an agent for soil improvement, remediation or protection against particular environmental pollution and used as an avenue for greenhouse gas mitigation [1].

When biochar is used as a soil-amendment material, it can affect the soil’s physical properties such as texture, porosity and pore-size distribution (PSD) because of its highly porous structure [2]. These changes then influence plant growth because the availability of water within the root zone is primarily determined by the physical properties of the soil around the roots. If biochar can improve soils’ water-retention, it may be possible to reduce the damage caused by drought [3].

Improving soils’ water-retention with biochar is partially dependent on the physical and chemical properties of the biochar, which are determined by the type of feedstock and pyrolysis processes used to produce the biochar and the amount of biochar added to the soil [4]. Certain feedstocks may be selected and the pyrolysis conditions may be tailored to produce biochars that maximise soils’ water-retention and storage [5]. Previous studies have investigated the water-retention capacity of biochar produced from crop residues and wood-based biomass feedstocks [3,4,5,6,7,8,9,10,11]; however, more information about the water-retention properties of biochar produced from various feedstocks is needed. Although it is generally known that pyrolysis temperature affects water-retention properties of biochar [3,4,5,11], the influence of pyrolysis temperatures can be different depending on the biomass feedstock. Therefore, the influence of feedstock and pyrolysis temperature on the water-retention property of biochar requires to be examined and clarified.

The PSD is known as one of the few important physical properties that affects a biochar’s water-retention property [4]. Biochar have a wide range of pore sizes from sub-nanometre to tens of micrometres [10]. Generally, pores in the nanometre size range are important for chemical interactions. However, those pores are not useful for plant-available water because plants are unable to overcome the high capillary forces that hold water in extremely small pores [10]. By contrast, pores in the micrometre-size range are useful for plant-available water.

Hydrophobicity is known as another important physicochemical properties that affect the water-retention property of a biochar [9]. A low pyrolysis temperature has been shown to increase the hydrophobicity of biochar and can limit their water-retention capacity [4,9]. Aliphatic compounds, which are abound with fresh biochar pyrolysed at low temperatures, are thought to cause hydrophobicity [9].

Thus, 21 types of biochar from seven different biomass feedstocks and at three pyrolysis temperatures were prepared. The prepared biochar’s water-retention related properties were evaluated via SEM micrography; hydrophobicity index (MED); PSD, which was measured by the mercury intrusion porosimetry (MIP) method; water-retention curve (WRC) and plant-available water capacity (AWC).

## 2. Materials and Methods

The selected biomass feedstocks included wood-chips [Japanese cedar (CE) and Japanese cypress (CY)], moso bamboo (MB) chips, rice husk (RH), sugarcane bagasse (SB), poultry manure (PM) and agricultural wastewater sludge (WS). These materials are generated in the rural areas of Japan and are excellent candidates for the potential use as biochar feedstocks. The CE, CY and MB were categorised as wood-based biomass, whereas RH and SB were categorised as crop residue. The PM was categorised as livestock manure, and WS was categorised as wastewater [12]. Chips of thinned CE and CY were purchased from a forestry cooperative. The CE and CY show a cuboid shape (length of long sides: 1–2 cm). The chip of thinned MB was purchased from a construction company. The MB shows a cuboid shape (length of long sides: 0.5–1 cm). The RH was collected in September from a rice processing facility in Ibaraki Prefecture, Japan. The RH shows a prolate spheroid shape (diameter of long sides: 0.7–1 cm). The SB was collected in March from a sugar factory in Kagoshima Prefecture, Japan. The SB shows a cylindrical shape (length of long sides: 1–2 cm). The mechanically dried PM was collected in September from a poultry farm in Ibaraki prefecture, Japan. The PM shows a granular shape (diameter: 0.1–0.2 cm). The mechanically dried WS was collected in November from a drainage facility for agricultural communities in Gumma Prefecture, Japan. The WS shows a granular shape (diameter: 0.5–0.7 cm). The biomass feedstocks were air-dried. The pretreatment was not conducted except for air-drying.

A heat-resistant container (50 cm × 50 cm × 15 cm) was filled 1/10 full with each feedstock. The mass of each feedstock in a container varied from 1 to 4 kg. Each container was placed in a temperature-controlled electric furnace (MB-202020-AC, Koyo Thermo Systems Co., Ltd., Nara, Japan). The reactor of the electric furnace shows a horizontal cylindrical shape (diameter 0.75 m; height 1 m). The reactor was purged with nitrogen gas in the beginning and sealed for pyrolysis. Then, the feedstock was pyrolyzed, heating at a rate of 2 °C min^−1^, and holding at 400 °C, 600 °C and 800 °C for two hours. After cooling, the produced biochar were homogenized by grounding and sieving to 0.5–2-mm-size fractions.

The volatile matter in the biochar were determined by weight loss after heating the biochar in a covered crucible at 950 °C for 6 min [13]. The ash content was determined by weight loss after combustion in air at 750 °C for 6 h [13]. The pH and electric conductivity (EC) of the biochar were measured in a 1:25 biochar:water suspension. Particle density was measured by the gas-displacement method using helium [14], and the mass fractions of C, H, N and S were measured using a 2400 Series II CHNS/O Elemental Analyzer (PerkinElmer, Inc., Waltham, MA, USA). The mass fraction of O was estimated by subtracting the mass fractions of the measured elements [i.e., O = 100 − (C + H + N + S + Ash content)].

Because hydrophobicity and PSD are important properties affecting water-retention in biochar, both parameters were investigated. The hydrophobicity indices of the prepared biochar samples were determined through the molarity-of-ethanol-droplet (MED) testing [15]. The hydrophobicities were classified by the value of ethanol concentration absorbed by biochar, and were categorised as hydrophilic [<1 M (mol L^−1^)], hydrophobic (1–2 M), strongly hydrophobic (2–3.5 M) or extremely hydrophobic (>3.5 M) [16]. Duplicate samples were used for those measurements.

The PSD of the biochar was measured by MIP (Pascal 140 and 440, Thermo Fisher Scientific Inc., Waltham, MA, USA). Using MIP, pressure was added in equal increments and the volume of intruding mercury was monitored at each pressure. The pressure ranges were 0.01–0.4 MPa for the Pascal 140 and 0.1–400 MPa for the Pascal 440, and the pore diameter corresponding to the additional pressure was approximated using the Washburn model, as expressed in Equation (1) [17]

(1) $$D=\frac{-4\sigma_{M}cos\theta }{P},$$

where *D* denotes the pore diameter (m), $\sigma_{M}$ is the surface tension of mercury (480 × 10^−6^ Pa m), *θ* represents the contact angle (140°) and *P* represents the additional pressure (Pa). Therefore, under the allocated pressure range, the pore diameter should range from 0.004 µm to 150 µm. The measurement of PSD using MIP was performed only once, considering the accuracy of the equipment as well as results reported in a previous study [7]. According to the definition provided by the Soil Science Society of America [18], pore sizes were classified into macropores (>75 µm), mesopores (30−75 µm), micropores (5−30 µm), ultra-micropores (0.1−5 µm) and cryptopores (<0.1 µm).

Plant-available water capacity (AWC) is the difference between the water content values at field capacity and the permanent wilting points [19]. Pressure ranges from −33 to −1500 kPa have often been suggested for AWC. Assuming the matric potential of the AWC ranges from −33 to −1500 kPa, the extreme diameters of the corresponding capillary pores were calculated as 0.2 µm and 9 µm. This calculation uses the capillary-rise equation [20], given by

(2) $$h=-\frac{2\sigma_{W}\cos\left(\alpha \right)}{\rho_{W}gr_{e}},$$

where *h* denotes the pressure head (m), $\rho_{W}$ is the water density (998 kg m^−3^ at 20 °C), $r_{e}$ is the capillary radius (m), *g* is gravitational acceleration (9.81 m s^−2^), $\sigma_{M}$ denotes the surface tension (kg s^−2^) and *α* is the angle of wetting (0°). Therefore, the pore size (0.2–9 μm) was classified as pores corresponding to the AWC.

Because pores in the micrometre-size range may play important roles in hydrologic processes [4,9,21], the micrometre pore structures of the prepared biochars were visualised using SEM. The measurements were performed on a JSM 5610 (JEOL, Tokyo, Japan). The scanning was performed at high accelerating voltage (15 kV). Micrographs were obtained at 500× and 150× magnification.

The WRC express the relationship between gravimetric water content and matric potential. Biochar samples were placed in cores (internal diameter of 5 cm and a height of 5.1 cm), which were set on a filter, and samples were then filled up to the upper end of the cores while being gently tapped. Treatments were performed in triplicates (*n* = 3). Core samples were saturated by applying zero water potential at half the sample height over a one-week period. The WRCs relate the gravimetric water content to the matric potential and are measured by the sandbox method [22] (for matric potentials greater than −3 kPa) or the pressure-plate method [19] (for matric potentials of −3 to −1500 kPa). The AWCs were calculated as the difference between the field capacity (water content at −33 kPa matric potential) and the wilting point (water content at −1500 kPa matric potential). After the WRCs were measured, the core samples were oven-dried to measure their bulk densities. The AWCs data were analyzed by a one-way analysis of variance (ANOVA) and Fisher’s least significant difference (LSD) test using the statistical application programming (BellCurve for Excel, Social Survey Research Information Co. Ltd., Tokyo, Japan). The differences between the means were examined with at a 0.05 probability level.

## 3. Results and Discussion

### 3.1. Physicochemical Properties of Biochar Samples

The measured physicochemical properties of the biochar samples are reported in Table 1. The biochar yields were influenced by the feedstock and pyrolysis temperature. The RH-, PM-, and WS-derived biochar yields (39–59%, 47–68% and 43–54%, respectively) were higher than those (22–41%, 23–39%, 25–39% and 19–28%, respectively) from the CE-, CY-, MB-, and SB-derived biochars, which may have been due to the higher ash content of RH, PM, and WS [23]. Moreover, the biochar yields decreased sharply as the pyrolysis temperature increased from 400 °C to 800 °C for all feedstocks. With an increase in pyrolysis temperature, the content of volatile matter decreased, and the ash content increased for most feedstocks. The hydrogen and oxygen content all decreased as pyrolysis temperature was increased, whereas the carbon content increased. These results are similar to those reported in previous studies [24,25,26]. The carbon contents were negatively correlated with the biochar yields, which indicating accelerated carbonization as the pyrolysis temperature increased [27]. The carbon contents of CE-, CY-, MB- and SB-derived biochar were larger than those in RH-, PM- and WS-derived biochar, which may have been due to the higher ash content of RH, PM, and WS. As reported in a previous study [28], the biochar from cow manure and wastewater sludge were lower carbon contents.

**Figure 1:**
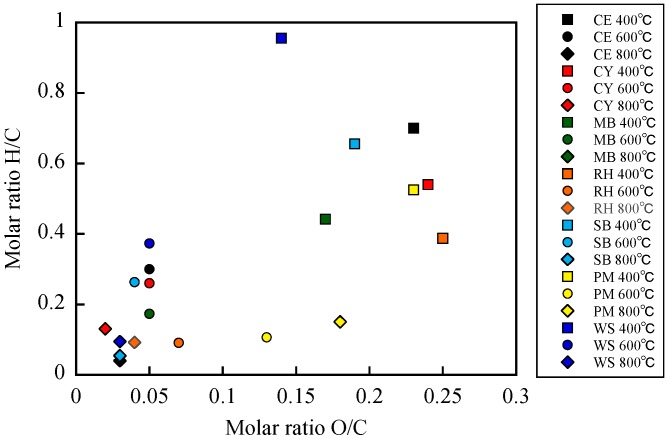
van Krevelin diagram of biochar samples (data taken from Table 1).

The van Krevelen diagram of biochar samples is shown in Figure 1. The molar H/C and O/C ratios were typically correlated with the degree of aromaticity and the degree of polarity, respectively [28]. The H/C and O/C ratios of biochar formed at 400 °C were higher than those of the biochar formed at 600 °C and 800 °C. The O/C and H/C ratios are correlated with surface hydrophilicity of biochar [29,30]. The lower O/C and H/C ratios for biochar formed at 600 °C and 800 °C indicate that the surfaces of biochar were more aromatic and less hydrophilic due to higher extent of carbonization and loss of polar functional groups at higher temperature [31,32]. In addition, the O/C and H/C ratios relate with stability of biochar against degradation in soils [33]. The lower O/C and H/C ratios for biochar formed at 600 °C and 800 °C could be higher stable. Schimmelpfennig and Glaser [33] recommended O/C ratio < 0.4 and H/C ratio < 0.6 as thresholds to identify the biochar stability. The H/C of the biochar formed from CE, SB and WS at 400 °C were 0.70, 0.66 and 0.96, respectively, and could be evaluated as lower stability.

Increases in particle density with increasing pyrolysis temperatures are consistent with results reported in previous studies [9,10,21]. Increases in particle density may be caused by the gradual condensation of carbon into aromatic structures and the concentration of ash in biochar [10].

The pHs of the biochar-water mixtures were alkaline for most feedstocks. Regardless of feedstock type, the pHs increased with higher pyrolysis temperatures, which is consistent with results reported in previous studies [24,25,26]. This can be attributed to the separation of the minerals from the organic matrix and the increase of ash content under high pyrolysis temperatures [34]. Similarly, the EC of the biochar-water mixtures, which is used to indicate the total water-soluble ions in biochar, also increased with higher pyrolysis temperatures, but varied very widely among the feedstocks. The increase in EC with increasing temperature was consistent with the increase in ash content, as suggested by a previous study [25]. The increase in pH and EC was primarily due to the release of certain basic elements (Ca^2+^ and Mg^2+^) as the pyrolysis temperature increased, as suggested in a previous study [25]. Therefore, the pH and EC values of the prepared biochars were correlated for all feedstocks (*r* = 0.7) [26].

### 3.2. Hydrophobicity Index of Biochar Samples

The biochar hydrophobicity index (MED value) was also dependent on the pyrolysis temperature (Table 1). The MED value of biochar formed at 400 °C was higher than that of the biochar formed at 600 °C and 800 °C, which was especially true for CE, SB and WS (Table 1). These results were consistent with previous studies, which suggested that high temperature leads to less hydrophobic biochar surfaces [4,9,35]. The MED of the biochar formed from CE, SB and WS at 400 °C were 2.8, 3.3 and 4.9 M, respectively, and were evaluated as strongly hydrophobic, strongly hydrophobic and extremely hydrophobic, also respectively. Those biochars showed higher molar ratio H/C (Figure 1). This result was consistent with previous studies, which suggested that the O/C and H/C ratios are correlated with surface hydrophilicity of biochar [29,30]. The MEDs of the biochar formed at 600 °C and 800 °C were less than 1.2 M and were classified as hydrophobic or hydrophilic for all types of biomass feedstock. These results demonstrated that biochar hydrophobicity became weak at pyrolysis temperatures greater than 600 °C. The differences in hydrophobicity could be explained by the changes in surface chemistry as a function of pyrolysis temperature [4,35].

### 3.3. Pore Structure of Biochar Samples

**Figure 2:**
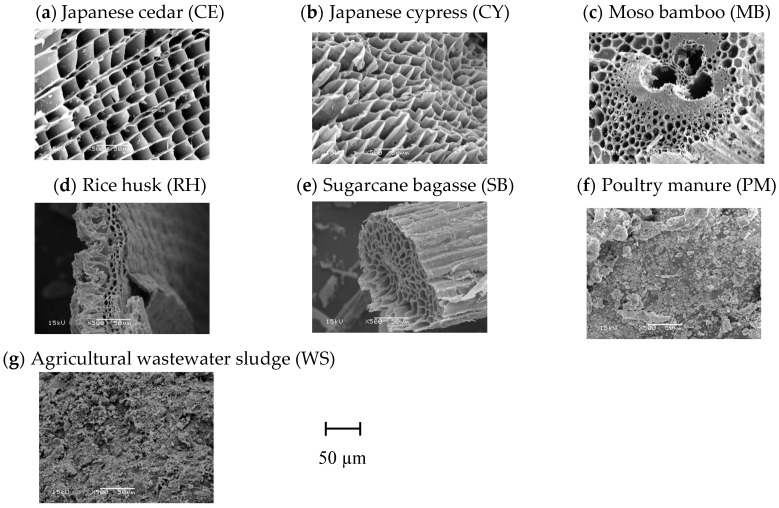
SEM micrographs of biochar samples formed at 600 °C. (**a**) Japanese cedar (CE); (**b**) Japanese cypress (CY); (**c**) moso bamboo (MB); (**d**) rice husk (RH); (**e**) sugarcane bagasse (SB); (**f**) poultry manure (PM); (**g**) agricultural wastewater sludge (WS).

In order to observe differences in pore structure between biochar produced from different feedstock, the SEM micrographs of the biochar formed at 600 °C are shown in Figure 2. Biochar produced from CE, CY, MB, RH and SB retained pore characteristics derived from the cellular structure of the plant-derived feedstocks. This is consistent with results reported in previous studies [7,21]. Contrarily, porous structures were not observed for biochars produced from PM and WS.

Biochar produced from CE and CY retained the regular cell-wall structure of their biomass feedstock with large pores in the order of 10 µm. The pore structure of biochar formed from MB was characterized by cell-wall structures with a wide range of sizes in the orders of 1–10 µm. The pore structure of biochar formed from RH and SB is characterized by cell-wall structures with sizes in the order of 1 µm.

Assuming that the matric potential of the AWC ranges from −33 to −1500 kPa, the extreme diameters of the corresponding capillary pores were calculated as 0.2 µm and 9 µm. For this reason, pores in the micrometre-size range may play an important role in promoting plant growth. Therefore, the pores in the micrometre-size range observed for biochar produced from CE, CY, MB, RH and SB may affect the water-retention capacity of biochar.

**Figure 3:**
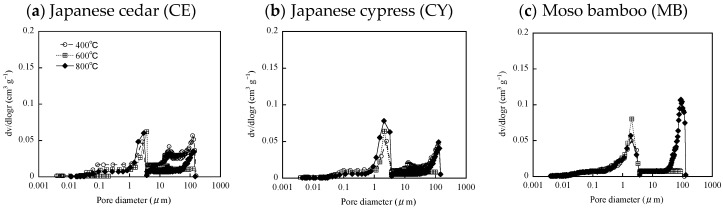

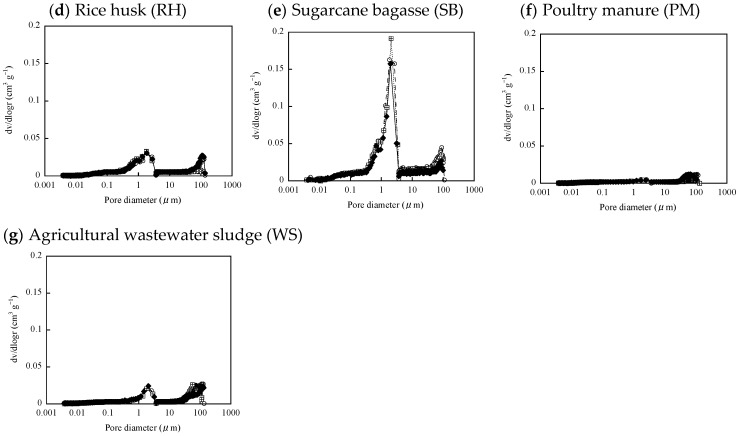
Pore size distribution (PSD) of biochar samples, measured by mercury-intrusion porosimetry (MIP). (**a**) Japanese cedar (CE); (**b**) Japanese cypress (CY); (**c**) moso bamboo (MB); (**d**) rice husk (RH); (**e**) sugarcane bagasse (SB); (**f**) poultry manure (PM); (**g**) agricultural wastewater sludge (WS).

The PSD of the biochar are shown in Figure 3. The PSDs of biochar formed with various pyrolysis temperatures were similar, which is consistent with results reported in previous studies [7]. Reportedly, pyrolysis temperature does not significantly affect the micrometre-range pore spaces of biochar [36]. In addition, previous studies have suggested that the majority of these pores are residual biological capillary structures of the raw-feedstock materials [21,37]. Therefore, the PSDs of biochar may depend on the pore structures of the feedstock.

When applying the MIP method, it is difficult to distinguish between intra and interparticle pores [10]: the PSDs measured by the MIP method involve both intraparticle and interparticle pores. Generally, the contribution of interparticle pores to pore volume is known to be larger with increasing pore diameter [9]. For pore diameters over 10 µm, pore volumes were different for the biochar formed at 400, 600 and 800 °C for most biomass feedstocks (Figure 3). These results might be due to the contribution associated with interparticle pores. As mentioned above, pores with diameters greater than 10 µm might not be important for water uptake by plants. Contrarily, a peak for intraparticle-pore diameter of around 2 µm would be important for the AWC (Figure 3). The peak height of around 2 µm for biochar derived from SB was the highest among all the biomass feedstocks. By contrast, the peak height at around 2 µm for biochar derived from PM was the lowest among all the biomass feedstocks. The peak height around 2 µm was similar for biochar derived from wood-based biomass (CE, CY and MB). Previous studies suggested that pore structures observed in SEM images corresponded to those measured by MIP [11]. However, in this study, there was not a clear relationship between SEM micrographs and PSDs (Figure 2 and Figure 3).

Volumes associated with different pore sizes and the volumes of pore size (0.2−9 µm) corresponding to the equivalent pore diameter with AWCs of the biochars are shown in Table 2. Differences in the volumes of macropore (>75 µm) and mesopore (30–75 µm) sizes among the biochars formed at 400, 600 and 800 °C are due to the contribution from interparticle pores, as mentioned above. As a result, total pore volumes varied considerably among biochars formed at 400, 600 and 800 °C. Micropore (5–30 µm) volumes of biochars derived from wood-chips (CE and CY) were larger than those of biochars derived from other feedstocks. However, micropore (5–30 µm) volumes of the biochars derived from CE, CY and SB clearly decreased with increasing pyrolysis temperatures. As volatiles are removed from the biomass during pyrolysis, shrinkage of feedstock particles occurs [38,39]. The shrinkage of feedstock increases with increasing pyrolysis temperatures [38]. Therefore, micropore (5–30 µm) volumes of biochars derived from CE, CY and SB may be affected by the shrinkage of feedstock.

Assuming that the matric potential of the AWC ranges from −33 to −1500 kPa, the extreme diameters of the corresponding capillary pores were calculated as 0.2 µm and 9 µm. For this reason, micropores and ultra-micropores are relatively important for enhancing soil’s water retention. Ultra-micropore (0.1–5 µm) volume of biochar derived from SB was the largest. Volumes of micropore (5–30 µm) and ultra-micropore (0.1–5 µm) sizes of biochar derived from PM and WS were relatively small. The relationship between pyrolysis temperatures and total pore volumes were not clear. Zhang and You [7] showed that volumes of micropore (5–30 µm) and ultra-micropore (0.1–5 µm) sizes of biochar derived from wood (poplar and pine) were 0.69–0.93 and 1.91–2.61 mL g^−1^, respectively. The micropores volumes were less than those of CE, CY and SB-derived biochar used in this study, while the ultra-micropore volumes were greater than those of biochar used in this study.

There was no clear relationship between pyrolysis temperature and pore volume corresponding to equivalent pore diameter with the AWCs of the biochars. The pore volumes corresponding to equivalent pore diameter with AWCs of biochar derived from SB was the largest among all feedstocks. This result is due to the micropore and ultra-micropore volumes of biochar derived from SB. The pore volumes of the biochars derived from wood-based biomass (CE, CY and MB) were relatively large. By contrast, the pore volumes of the biochars derived from PM and WS were relatively small. This result indicated that biochars derived from PM and WS are not suitable as a potential amendment for improving soils’ water-retention.

### 3.4. Water-Retention of Biochar Samples

**Figure 4:**
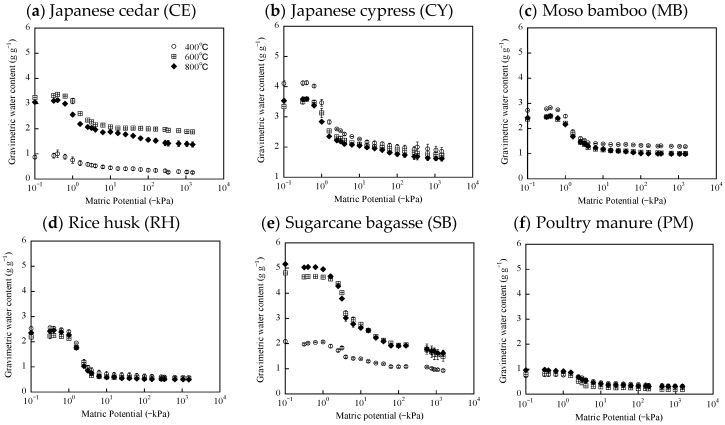

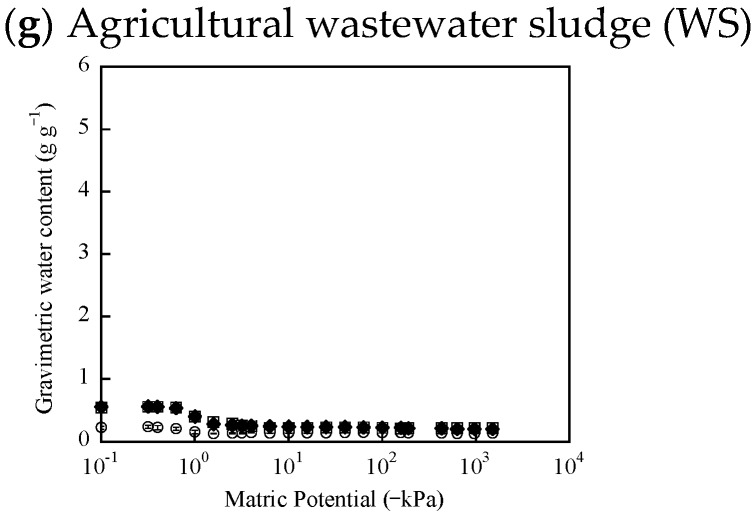
Water retention curves (WRCs) of biochar samples. (**a**) Japanese cedar (CE); (**b**) Japanese cypress (CY); (**c**) moso bamboo (MB); (**d**) rice husk (RH); (**e**) sugarcane bagasse (SB); (**f**) poultry manure (PM); (**g**) agricultural wastewater sludge (WS).

The WRCs and AWCs of the biochar samples are shown in Figure 4 and Table 3, respectively. The WRC of the biochars produced from CE, SB and WS was different for different pyrolysis temperatures (Figure 4). The biochars formed at 400 °C exhibited strong or extremely hydrophobic properties (Table 1). Therefore, the WRCs of the biochars formed from CE, WS and SB at 400 °C would be influenced by their strong water-repellent properties. In addition, the AWCs of the biochars formed from CE and SB at 400 °C were significantly less than those formed at 600 °C and 800 °C (Table 3). These results suggest that the strong water-repellent properties of these biochars negatively influenced WRC and AWC for raw biochar samples. The AWC of raw biochars would be substantially affected by its hydrophobicity. However, previous study suggested that biochar hydrophobicity cannot have an effect on soils’ WRC and AWC after soil incubation [3]. Therefore, the hydrophobicity of raw biochar may be reduced in soils. The WRC and AWC of biochars derived from other feedstocks did not concededly differ with pyrolysis temperature (Figure 4 and Table 3). This result could be due to the weak hydrophobicity of those biochar formed at 400 °C (Table 1). In addition, this result is consistent with the PSD measurements, which were similar for various pyrolysis temperatures for each biochar (Figure 3).

The AWCs of biochars produced from CE, CY and SB at 600 and 800 °C were greater than those of biochars produced from other feedstocks at 600 and 800 °C (Table 3). This tendency is consistent with the degree of pore volume corresponding to equivalent pore diameter with AWC (Table 2). From the experimental results, we can suggest that the biochars derived from wood-chips (CE and CY) and SB have greater potential for enhancing soils’ water-retention. However, raw biochars were used in this study. Therefore, measurements of water retention properties using soils amended with those biochars are needed to demonstrate soil improvement effects in the future.

**Figure 5:**
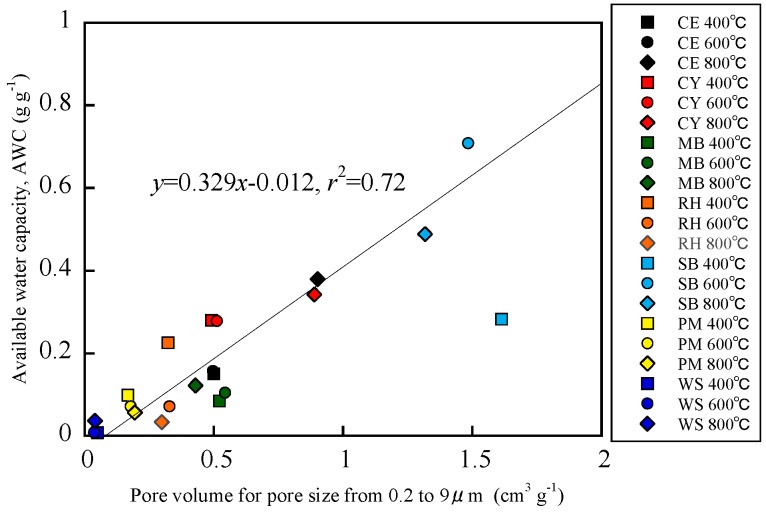
Relationship between volumes for pore sizes from 0.2 to 9 µm, estimated from pore size distribution (PSD) measured by the mercury intrusion porosimetry (MIP) method, and available water capacity, estimated from water retention curves (WRCs), of biochar samples.

The relationship between pore volumes corresponding to equivalent pore diameters with AWC and AWC is shown in Figure 5. Linear regression analysis was conducted for the relationship using data from all biochar with Microsoft Excel and the regression line is also shown in the Figure 5. Most data points from the biochars were plotted along the regression line. However, the data point from the SB-derived biochar formed at 400 °C were plotted below the straight line. The biochar exhibited strong hydrophobicity (Table 1). This result suggests that the AWC of biochars can be roughly estimated from the PSD measured by MIP. Generally, the WRC measurements are time-consuming (over a few months). Therefore, the pore volumes corresponding to equivalent pore diameter with AWC measured by MIP may serve as a useful index to seek a potential amendment for enhancing soils’ water-retention.

## 4. Conclusions

In this study, we clarified the influence of feedstock and pyrolysis temperature on the water-retention related properties of biochar. Wood-chips (CE and CY), MB, RH, SB, PM and WS were each pyrolysed at 400, 600 and 800 °C with a retention time of two hours. The SEM micrograph, PSD, hydrophobicity index, WRC and AWC of the biochar were examined.

Hydrophobicity indices of the biochars formed from CE, SB and WS at 400 °C were high. The biochar showed higher molar ratio H/C, indicating lower stability against degradation in soils. As the pyrolysis temperature was increased, the hydrophobicity index and molar ratio H/C decreased. From the point of hydrophobicity and stability, biochar produced at higher temperature may be recommended as soil amendment. However, more energy is required for pyrolysis at higher temperatures. Therefore, further investigation can be needed to select optimal pyrolysis temperature.

The PSD of the biochars was not significantly affected by pyrolysis temperatures. The AWCs of biochars derived from PM and WS were relatively small due to poor pore structures. This result shows that biochars derived from PM and WS are not suitable as a potential amendment for enhancing soils’ water retention either. The AWCs of biochars formed from CE, CY and SB at 600 and 800 °C were greater than those of biochars formed from other feedstocks at 600 and 800 °C. This tendency is consistent with the degree of pore volume for pore size corresponding to AWC, measured by MIP. In addition, volumes of pores corresponding to equivalent pore diameter with AWC measured by the MIP method may be a useful index for seeking as a potential amendment for enhancing soils’ water-retention.

From the experimental results, we can suggest that the biochars derived from wood-chips (CE and CY) and SB have greater potential for enhancing soils’ water-retention. However, raw biochars were used in this study. Therefore, measurements of water retention capacities using soils amended with those biochars for not only laboratory but also field will be needed to demonstrate soil improvement effects in the future.

## Figures and Tables

**Table 1:** Physicochemical properties of biochar samples.

Feedstock	Pyrolysis Temperature	Biochar Yield	Volatile Matter	Ash	C	H	N	S	O	Particle Density	pH	EC	MED
	°C	%w/w	%w/w	%w/w	%w/w	%w/w	%w/w	%w/w	%w/w	mg m^−3^	–	dS m^−1^	mol L^−1^
**Wood-based Biomass**													
Japanese cedar (CE)	400	41	58	0.1	72.0	4.2	1.6	0.0	22.1	1.30	7.8	0.2	2.8
	600	28	26	1.7	87.7	2.2	2.4	0.0	6.0	1.45	8.8	0.4	0.7
	800	22	16	2.6	90.5	0.3	3.1	0.0	3.5	1.71	8.4	1.7	0.0
Japanese cypress (CY)	400	39	48	2.1	71.2	3.2	0.7	0.0	22.8	1.40	5.4	0.2	0.8
	600	28	20	2.8	87.3	1.9	1.6	0.0	6.4	1.52	83	0.4	0.5
	800	23	10	4.0	91.8	1.0	1.0	0.0	2.2	1.81	9.1	0.5	0.2
Moso bamboo (MB)	400	28	40	6.1	73.3	2.7	1.3	0.0	16.6	1.26	7.4	1.9	0.3
	600	28	29	8.3	83.2	1.2	2.1	0.0	5.2	1.63	10.3	3.9	0.0
	800	25	26	6.7	88.1	0.4	1.0	0.0	3.8	1.65	9.7	7.0	0.0
**Agricultural Residue**													
Rice husk (RH)	400	59	38	47.9	37.2	1.2	1.3	0.0	12.4	1.60	6.7	0.7	0.2
	600	48	27	54.9	39.5	0.3	1.9	0.0	3.4	1.69	10.2	1.0	0.2
	800	39	11	57.7	39.0	0.3	1.0	0.0	2.0	1.74	10.4	1.6	0.0
Sugarcane bagasse (SB)	400	38	56	12.4	65.4	3.6	1.0	1.3	16.3	1.16	5.0	0.2	3.3
	600	22	34	18.6	75.3	1.7	0.7	0.0	3.8	1.36	7.8	0.2	1.2
	800	19	22	16.1	79.4	0.4	0.7	0.0	3.6	1.41	9.8	0.2	1.5
**Livestock Manure**													
Poultry manure (PM)	400	68	28	48.4	34.3	1.5	5.1	0.0	10.7	1.71	10.8	10.2	1.3
	600	62	17	56.7	33.8	0.3	3.7	0.0	5.5	1.73	12.0	18.8	0.0
	800	47	12	68.2	23.9	0.3	2.2	0.0	5.4	1.78	12.2	26.5	0.0
**Wastewater**													
Agricultural wastewater	400	54	35	37.1	42.7	3.4	8.1	0.6	8.1	1.53	7.3	0.2	4.9
Sludge (WS)	600	46	11	52.0	38.6	1.2	5.8	0.0	2.4	1.89	8.3	0.3	0.0
	800	43	5	57.0	37.9	0.3	3.4	0.0	1.4	2.09	8.0	0.3	0.0

**Table 2:** Volumes of different pore size and pores corresponding to available water capacity (AWC) of biochar samples, estimated from pore-size distribution (PSD) measured by mercury-intrusion porosimetry (MIP) method.

Feedstock	Pyrolysis Temperature	Total Volume	Macropores (> 75 μm)	Mesopores (30–75 μm)	Micropores (5–30 μm)	Ultra-Micropores (0.1–5 μm)	Cyptopores (<0.1 μm)	Pores Corresponds to Available Water Capacity (0.2–9 μm)
	°C	(cm^3^ g^−1^)	(cm^3^ g^−1^)	(cm^3^ g^−1^)	(cm^3^ g^−1^)	(cm^3^ g^−1^)	(cm^3^ g^−1^)	(cm^3^ g^−1^)
**Wood-based Biomass**								
Japanese cedar (CE)	400	4.09	0.43	0.88	2.51	0.22	0.06	0.50
	600	1.63	0.03	0.16	1.16	0.22	0.05	0.50
	800	1.85	0.27	0.25	0.95	0.36	0.03	0.90
Japanese cypress	400	2.67	0.35	0.42	1.65	0.19	0.06	0.49
(CY)	600	1.71	0.02	0.12	1.32	0.21	0.05	0.51
	800	1.74	0.32	0.26	0.49	0.65	0.02	0.89
Moso bamboo (MB)	400	0.99	0.01	0.07	0.22	0.54	0.15	0.52
	600	0.99	0.01	0.06	0.20	0.54	0.18	0.54
	800	1.30	0.17	0.24	0.20	0.25	0.44	0.43
**Agricultural Residue**								
Rice husk (RH)	400	0.84	0.15	0.16	0.13	0.33	0.08	0.32
	600	0.57	0.03	0.04	0.08	0.33	0.08	0.33
	800	0.87	0.20	0.18	0.12	0.31	0.07	0.30
Sugarcane bagasse	400	3.23	0.22	0.53	1.23	1.15	0.11	1.61
(SB)	600	2.91	0.19	0.41	1.04	1.12	0.14	1.48
	800	2.71	0.38	0.35	0.89	0.92	0.17	1.32
**Livestock Manure**								
Poultry manure (PM)	400	1.07	0.25	0.37	0.21	0.15	0.09	0.17
	600	1.11	0.12	0.52	0.23	0.16	0.08	0.18
	800	1.12	0.18	0.34	0.32	0.17	0.11	0.19
**Wastewater**								
Agricultural	400	0.49	0.10	0.18	0.09	0.04	0.09	0.05
wastewater	600	0.26	0.02	0.07	0.03	0.04	0.10	0.04
Sludge (WS)	800	0.52	0.06	0.27	0.06	0.04	0.09	0.04

**Table 3:** Available water capacity (AWC) estimated from water retention curves (WRC) of biochar samples.

Feedstock	Pyrolysis Temperature	Available Water Capacity (AWC)
	°C	(g g^−1^)
**Wood-based Biomass**		
Japanese cedar (CE)	400	0.12 (0.03) efg
	600	0.18 (0.03) defg
	800	0.38 (0.10) bc
Japanese cypress (CY)	400	0.28 (0.07) bcdef
	600	0.28 (0.00) bcdef
	800	0.34 (0.11) bcd
Moso bamboo (MB)	400	0.08 (0.01) g
	600	0.10 (0.03) efg
	800	0.12 (0.03) efg
**Agricultural Residue**		
Rice husk (RH)	400	0.11 (0.06) defg
	600	0.07 (0.00) g
	800	0.03 (0.01) g
Sugarcane bagasse (SB)	400	0.28 (0.02) cde
	600	0.71 (0.43) a
	800	0.49 (0.15) b
**Livestock Manure**		
Poultry manure (PM)	400	0.10 (0.03) fg
	600	0.07 (0.01) g
	800	0.06 (0.01) g
**Wastewater**		
Agricultural wastewater	400	0.01 (0.00) g
Sludge (WS)	600	0.01 (0.01) g
	800	0.04 (0.00) g

Values are means (standard deviation) (*n* = 3). Means with the same letter are not significantly different from each other (*P* < 0.05).

## References

[1] Lehmann J., Joseph S. (2013). Biochar for Environmental Management: Science, Technology and Implementation.

[2] Jien S., Ok Y.S., Tsanget D.C.W., Bolan J., Novak J.M. (2018). Physical Characteristics of Biochars and Their Effects on Soil Physical Properties. Biochar from Biomass and Waste. Fundamentals and Applications.

[3] Kameyama K., Miyamoto T., Iwata Y., Shiono T. (2016). Effects of biochar produced from sugarcane bagasse at different pyrolysis temperatures on water retention of a calcaric dark red soil. Soil Sci..

[4] Kinney T.J., Masiello C.A., Dugan B., Hockaday W.C., Dean M.R., Zygourakis K., Barnes R.T. (2012). Hydrologic properties of biochars produced at different temperatures. Biomass Bioenergy.

[5] Novak J.M., Busscher W.J., Watts D.W., Amonette J., Ippolito J.A., Lima I.M., Gaskin J., Das K.C., Steiner C., Ahmedna M. (2012). Biochars impact on soil moisture storage in an Ultisol and two Aridisols. Soil Sci..

[6] Mollinedo J., Schumacher T.E., Chintala R. (2015). Influence of feedstocks and pyrolysis on biochar’s capacity to modify soil water retention characteristics. J. Anal. Appl. Pyrol..

[7] Zhang J., You C. (2013). Water holding capacity and absorption properties of wood chars. Energy Fuels.

[8] Liu Z., Dugan B., Masiello C.A., Gonnermann H.M. (2017). Biochar particle size, shape and porosity act together to influence soil water properties. PLoS ONE.

[9] Gray M., Johnson M.G., Dragila M.I., Kleber M. (2014). Water uptake in biochars: The roles of porosity and hydrophobicity. Biomass Bioenergy.

[10] Brewer C.E., Chuang V.J., Masiello C.A., Gonnermann H., Gao X., Dugan B., Driver L.E., Panzacchi P., Zygourakis K., Davies C.A. (2014). New approaches to measuring biochar density and porosity. Biomass Bioenergy.

[11] Břendová K., Száková J., Lhotka M., Krulikovská T., Punčochář M., Tlustoš P. (2017). Biochar physicochemical parameters as a result of feedstock material and pyrolysis temperature: predictable for the fate of biochar in soil?. Environ. Geochem. Health.

[12] NEDO (2014). Renewable Energy Technology White Paper.

[13] (2007). Standard Test Method for Chemical Analysis of Wood Charcoal.

[14] Gao X., Masiello C.A., Singh B., Camps-Arbestain M., Lehmann J. (2017). Analysis of biochar porosity by pycnometry. Biochar: A Guide to Analytical Methods.

[15] King P.M. (1981). Comparison of methods for measuring severity of water repellence of sandy soils and assessment of some factors that affect its measurement. Aust. J. Soil Res..

[16] Johnson M.S., Lehmann J., Steenhuis T.S., de Oliveira L.V., Fernandes E.C.M. (2005). Spatial and temporal variability of soil water repellency of Amazonian pastures. Aust. J. Soil Res..

[17] Washburn E.W. (1921). Note on a method of determining the distribution of poresizes in a porous material. PNAS.

[18] Soil Science Society of America (1997). Glossary of Soil ScienceTerms.

[19] Dane J.H., Hopmans J.W., Dane J.H., Topp C. (2002). Pressure plate extractor. Methods of Soil Analysis. Part 4. Physical Methods.

[20] Jury W.A., Horton R. (2004). Soil Physics.

[21] Sun H., Hockaday W.C., Masiello C.A, Zygourakis K. (2012). Multiple controls on the chemical and physical structure of biochars. Ind. Eng. Chem. Res..

[22] Jamison V.C. (1958). Sand-silt suction column for determination of moisture retention. Soil Sci. Soc. Am. Proc..

[23] Windeatt J.H., Ross A.B., Williams P.T., Forster P.M., Nahil M.A., Singh S. (2014). Characteristics of biochars from crop residues: Potential for carbon sequestration and soil amendment. J. Environ. Manag..

[24] Suliman W., Harsh J.B., Abu-Lail N.I., Fortuna A.-M., Dallmeyer I., Garcia-Perez M. (2016). Influence of feedstock source and pyrolysis temperature on biochar bulk and surface properties. Biomass Bioenergy.

[25] Rehrah D., Bansode R.R., Hassan O., Ahmedna M. (2016). Physico-chemical characterization of biochars from solid municipal waste for use in soil amendment. J. Anal. Appl. Pyro..

[26] Kameyama K., Iwata Y., Miyamoto T. (2017). Biochar Amendment of Soils According to their Physicochemical Properties. JARQ.

[27] Al-Wabel M.I., Al-Omran A., El-Naggar A.H., Nadeem M., Usman A.R.A. (2013). Pyrolysis temperature induced changes in characteristics and chemical composition of biochar produced from conocarpus wastes. Bioresour. Technol..

[28] Sun X., Shan R., Li X., Pan J., Liu X., Deng R., Song J. (2017). Characterization of 60 types of Chinese biomass waste and resultant biochars in terms of their candidacy for soil application. GCB Bioenergy.

[29] Chun Y., Sheng G., Chiou C.T., Xing B. (2004). Compositions and sorptive properties of crop residue-derived chars. Environ. Sci. Technol..

[30] Chen X., Chen G., Chen L., Chen Y., Lehmann J., McBride M.B., Hay A.G. (2011). Adsorption of copper and zinc by biochars produced from pyrolysis of hardwood and corn straw in aqueous solution. Bioresour. Technol..

[31] Chen Z., Chen B., Zhou D., Chen W. (2012). Bisolute sorption and thermodynamic behavior of organic pollutants to biomass-derived biochars at two pyrolytic temperatures. Environ. Sci. Technol..

[32] Ahmad M., Lee S.S., Dou X., Mohan D., Sung J.K., Yang J.E., Ok Y.S. (2012). Effects of pyrolysis temperature on soybean stover-and peanut shell-derived biochar properties and TCE adsorption in water. Bioresour. Technol..

[33] Schimmelpfennig S., Glaser B. (2012). One Step Forward toward Characterization: Some Important Material Properties to Distinguish Biochars. J. Environ. Qual..

[34] Novak J.M., Lima I., Xing B., Gaskin J.W., Steiner C., Das K.C., Ahmedna M., Rehrah D., Watts D.W., Busscher W.J., Schomberg H. (2009). Characterization of Designer Biochar Produced at Different Temperatures and Their Effects on a Loamy Sand. Ann. Environ. Sci..

[35] Suliman W., Harsh J.B., Abu-Lail N.I., Fortuna A., Dallmeyer I., Garcia-Pérez M. (2017). The role of biochar porosity and surface functionality in augmenting hydrologic properties of a sandy soil. Biomass Bioenergy.

[36] Hyväluoma J., Hannula M., Arstila K., Wang H., Kulju S., Rasa K. (2018). Effects of pyrolysis temperature on the hydrologically relevant porosity of willow biochar. J. Anal. Appl. Pyro..

[37] Wildman J., Derbyshire F. (1991). Origins and functions of macroporosity in activated carbons from coal and wood precursors. Fuel.

[38] Emmerich F.G., Luengo C.A. (1996). Babassu charcoal: A sulfurless renewable thermo-reducing feedstock for steelmaking. Biomass Bioenergy.

[39] Freitas J.C.C., Cunha A.G., Emmerich F.G. (1997). Physical and chemical properties of a Brazilian peat char as a function of HTT. Fuel.

